# ^3^He spin-echo scattering indicates hindered diffusion of isolated water molecules on graphene-covered Ir(111)

**DOI:** 10.3389/fchem.2023.1229546

**Published:** 2023-10-06

**Authors:** Signe Kyrkjebø, Andrew Cassidy, Sam Lambrick, Andrew Jardine, Bodil Holst, Liv Hornekær

**Affiliations:** ^1^ Center for Interstellar Catalysis, Department of Physics and Astronomy, Aarhus University, Aarhus, Denmark; ^2^ Interdisciplinary Nanoscience Center, Aarhus University, Aarhus, Denmark; ^3^ Cavendish Laboratory, University of Cambridge, Cambridge, United Kingdom; ^4^ Institute of Physics and Technology, University of Bergen, Bergen, Norway

**Keywords:** surface dynamics, surface diffusion, wettability, helium-3 spin-echo scattering, graphene, iridium

## Abstract

The dynamics of water diffusion on carbon surfaces are of interest in fields as diverse as furthering the use of graphene as an industrial-coating technology and understanding the catalytic role of carbon-based dust grains in the interstellar medium. The early stages of water–ice growth and the mobility of water adsorbates are inherently dependent on the microscopic mechanisms that facilitate water diffusion. Here, we use ^3^He spin-echo quasi-inelastic scattering to probe the microscopic mechanisms responsible for the diffusion of isolated water molecules on graphene-covered and bare Ir(111). The scattering of He atoms provides a non-invasive and highly surface-sensitive means to measure the rate at which absorbates move around on a substrate at very low coverage. Our results provide an approximate upper limit on the diffusion coefficient for water molecules on GrIr(111) of $<10^{-12}$ m^2^/s, an order of magnitude lower than the coefficient that describes the diffusion of water molecules on the bare Ir(111) surface. We attribute the hindered diffusion of water molecules on the GrIr(111) surface to water trapping at specific areas of the corrugated moiré superstructure. Lower mobility of water molecules on a surface is expected to lead to a lower ice nucleation rate and may enhance the macroscopic anti-icing properties of a surface.

## 1 Introduction

The microscopic mechanisms that facilitate water transport on carbon surfaces are not well understood (Bartels-Rausch, 2013; Bui et al., 2023) despite water diffusion on carbon playing a role in a wide range of fields, including material science and astrochemistry (Shavlov et al., 2007; Hama and Watanabe, 2013; Schertzer and Iglesias, 2018). Graphene, a 2D array of sp^2^-hybridized carbon atoms, has attracted interest as a potential anti-corrosion (Kyhl et al., 2015; Yu et al., 2018; Camilli et al., 2019) or anti-icing coating (Akhtar et al., 2019; Kyrkjebø et al., 2021). Water molecules on a surface may react with surface atoms, contributing to corrosion, or cause friction and wear. By understanding the principles of molecular diffusion in more detail, it may be possible to develop more effective strategies to control these processes. In the interstellar medium, the freeze-out of water molecules onto the surfaces of dust grains, some of which are made from carbonaceous materials, provides chemical repositories for increasing molecular complexity. Water–ice-covered dust grains act as seeds for the formation of complex organic molecules, boosting interstellar chemical complexity (Hama and Watanabe, 2013; Van Dishoeck et al., 2013; Fulvio et al., 2021). To understand the role of the carbon surface in these processes, it is crucial to consider the initial stages of ice nucleation and crystalline growth, and the diffusion of water molecules on carbon surfaces is key to both.

Macroscopically, the ability of a surface to maintain contact with water, commonly referred to as wetting, can be measured as the contact angle of an equilibrated liquid water droplet placed on the surface. The ability of water to wet a layer of graphene has been intensively studied over the last decade, with a large variation in the reported water contact angles, ranging from (42 ± 3)° (Prydatko et al., 2018) to (127 ± 4)° (Wang et al., 2009). While external parameters like contamination, environmental effects, and graphene synthesis differences contribute to the discrepancies reported in the literature, the measured contact angle is highly dependent on the substrate used (Rafiee et al., 2012; Li et al., 2013; Raj et al., 2013; Taherian et al., 2013; Parobek and Liu, 2015; Belyaeva and Schneider, 2020). Theoretical studies predict that the contact angle of graphene is controlled by the balance of polarization at the graphene–water interface and polarization at the graphene–substrate interface (Shih et al., 2013; Kong et al., 2018).

Microscopic measurements of the water–surface interaction focus on the adsorption and desorption kinetics of water molecules, utilizing experimental techniques such as low-temperature scanning tunneling microscopy (LT-STM) (Standop et al., 2015), temperature-programmed desorption (TPD) (Souda, 2012; Smith et al., 2014), and helium atom scattering (HAS). HAS is a technique particularly suitable for studying the microscopic morphology, structure, and dynamics of water (Daschbach et al., 2004; Andersson et al., 2007). As a surface probe, helium atoms are chemically inert and uncharged, and scatter from the outermost electron density distribution of the surface atoms. This makes HAS a non-destructive and highly surface-sensitive technique that can be used to study the properties of water on a surface without altering its structure or behavior. The cross-section for helium atoms to scatter from a single adsorbate is large, typically approaching 120 Å^2^ (Farias and Rieder, 1998), making helium atoms particularly suitable for studying adsorbate behavior at low coverages in the single molecule diffusion regime.

Helium atoms may scatter elastically from static surface atoms or quasi-elastically from moving adsorbates. In the energy transfer spectrum, quasi-elastic scattering from moving adsorbates will contribute to a broadening around the elastic peak, which gives information on the dynamics of the moving adsorbates on the surface. Helium-3 spin-echo scattering (^3^HeSE) significantly increases the energy resolution of He scattering techniques by avoiding the use of time-of-flight measurements to detect quasi-elastic scattering losses (Jardine et al., 2009). In ^3^HeSE, incoming helium atoms are spin-polarized and split into two spin components using a magnetic field. One of the spin components is accelerated, while the other is deaccelerated, separating the atoms in the so-called “spin-echo time,” *τ*_SE_. The spin-encoded components scatter from the surface and are subsequently recombined. The polarized spin of the scattered beam is then measured as a function of *τ*_SE_, providing a measure of the loss in the correlation of the spin-encoded beam as it scatters from the surface. Aperiodic motion on the surface, such as a diffusing adsorbate, will give rise to quasi-elastic scattering, leading to a loss in correlation, which is measured as an exponential decay in the polarized signal of scattered ^3^HeSE atoms.

^3^HeSE has recently been used to measure the diffusion properties of molecular water on a range of surfaces (Tamtögl et al., 2020; Tamtögl et al., 2021). Tamtögl et al. (2021) studied the diffusion of single water molecules on graphene prepared on Ni(111). They demonstrated that single water molecules jump from the center of one hexagon in graphene to the center of another, with a tracer diffusion coefficient of (4.1 ± 0.2) × 10^−10^ m^2^/s and an activation barrier of (60 ± 4) meV. Graphene prepared on Ni(111) is a strongly coupled system that, due to almost identical lattice constants, results in a relatively flat surface energy landscape (Batzill, 2012). Whether these results correlate with water diffusion on other graphene-supported systems is the focus of the current study.

In this study, we use ^3^HeSE measurements to investigate the diffusion properties of water molecules on Ir(111) and graphene prepared on Ir(111). The Ir(111) substrate is used because, in contrast to GrNi(111), GrIr(111) is a weakly bound system in which graphene can be considered to be quasi-free-standing (Busse et al., 2011; Batzill, 2012). The slight mismatch in lattice constant between the graphene unit cell and the Ir(111) unit cell gives rise to a moiré superstructure. The results are compared to the previous measurements performed on graphene on Ni(111).

## 2 Materials and methods

The ^3^HeSE instrument at the Cavendish Laboratory, University of Cambridge, was used for He scattering measurements (Jardine et al., 2009). The instrument consists of an ultra-high-vacuum chamber with a base pressure below 5 × 10^−11^ mbar. The sample sits at the end of a cryo-finger cooled with liquid nitrogen, where a filament allows for sample heating. The sample is interrogated by a 2 mm-focused beam of ^3^He atoms, which arrives at the sample via supersonic expansion through a nozzle cooled via a closed-cycle He compressor, giving the ^3^He atoms a nominal kinetic energy of 8 meV corresponding to a wavevector of 3.4 Å^−1^ (Jardine et al., 2009). The focused ^3^He beam is scattered from the sample, and scattered atoms are detected. The source-detector angle is fixed at 44.4°, and the scattering angle is changed by rotating the sample with 3 rotational degrees of freedom. For the ^3^HeSE experiments, the incoming ^3^He atoms are nuclear spin-polarized in a magnetic field; the polarized atom beam then enters a solenoid, where the magnetic field encodes nuclear spin. This beam of spin-polarized, spin-encoded ^3^He atoms scatters from the sample before passing through an identical but sign-reversed magnetic field at a second solenoid, where spins are decoded and spin-analyzed before reaching the detector. The temporal window of the instrument is between sub-picoseconds and 2 nanoseconds (Jardine et al., 2009). Further details about the instrument are provided elsewhere (Alexandrowicz and Jardine, 2007; Jardine et al., 2009). Scanning tunneling microscopy (STM) images were recorded in separate ultra-high-vacuum chambers at the Center for Interstellar Catalysis, Aarhus University. The pristine Ir(111) surface was characterized using a CreaTec STM at LN_2_ temperatures, and the GrIr(111) was characterized using an Aarhus-type STM at room temperature. STM images were analyzed using WSxM software (Horcas et al., 2007).

The sample is a 3 mm-thick Ir single crystal with a diameter of 7 mm, polished at the (111) surface (±0.1°). This Ir(111) surface was cleaned, *in situ*, at the characterization chamber, with several cycles of Ar^+^ sputtering and annealing, followed by annealing in an oxygen atmosphere. Graphene sheets were prepared on the clean Ir(111) via a combination of temperature-programmed growth and chemical vapor deposition (Coraux et al., 2009). The substrate was exposed to ethylene gas at room temperature and a partial pressure of 2 × 10^−7^ mbar for 15 min. The gas was pumped away, and the sample was flashed to 1,180°C and then cooled to 900°C in an ethylene partial pressure of 8 × 10^−7^ mbar for 15 min.

For water adsorption experiments, deionized water was purified via several freeze–pump–thaw cycles. Water was deposited onto LN_2_-cooled Ir(111) and GrIr(111) samples via chamber backfilling using a needle valve to achieve the required partial pressure in the sample chamber. To measure water diffusion via ^3^HeSE measurements, water was adsorbed on the substrate at 120 K while the elastically scattered helium reflectivity signal was monitored. 0.1 and 0.25 L water was deposited on Ir(111) and GrIr(111), respectively, which, in both cases, resulted in a 60% attenuation of the helium reflectivity signal. An attenuation of 75% of the helium signal was recently estimated to correspond to a coverage of 0.07 ML on GrNi(111) (Tamtögl et al., 2021), and we use this as a rough estimation for the coverage obtained on Ir(111) and GrIr(111). It should be noted that we do not expect our analysis and conclusions to be sensitive to the actual coverage achieved. This sample was then interrogated by the spin-polarized, spin-encoded beam of ^3^He atoms, and the scattering signal was recorded.

## 3 Results and analysis

**FIGURE 1:**
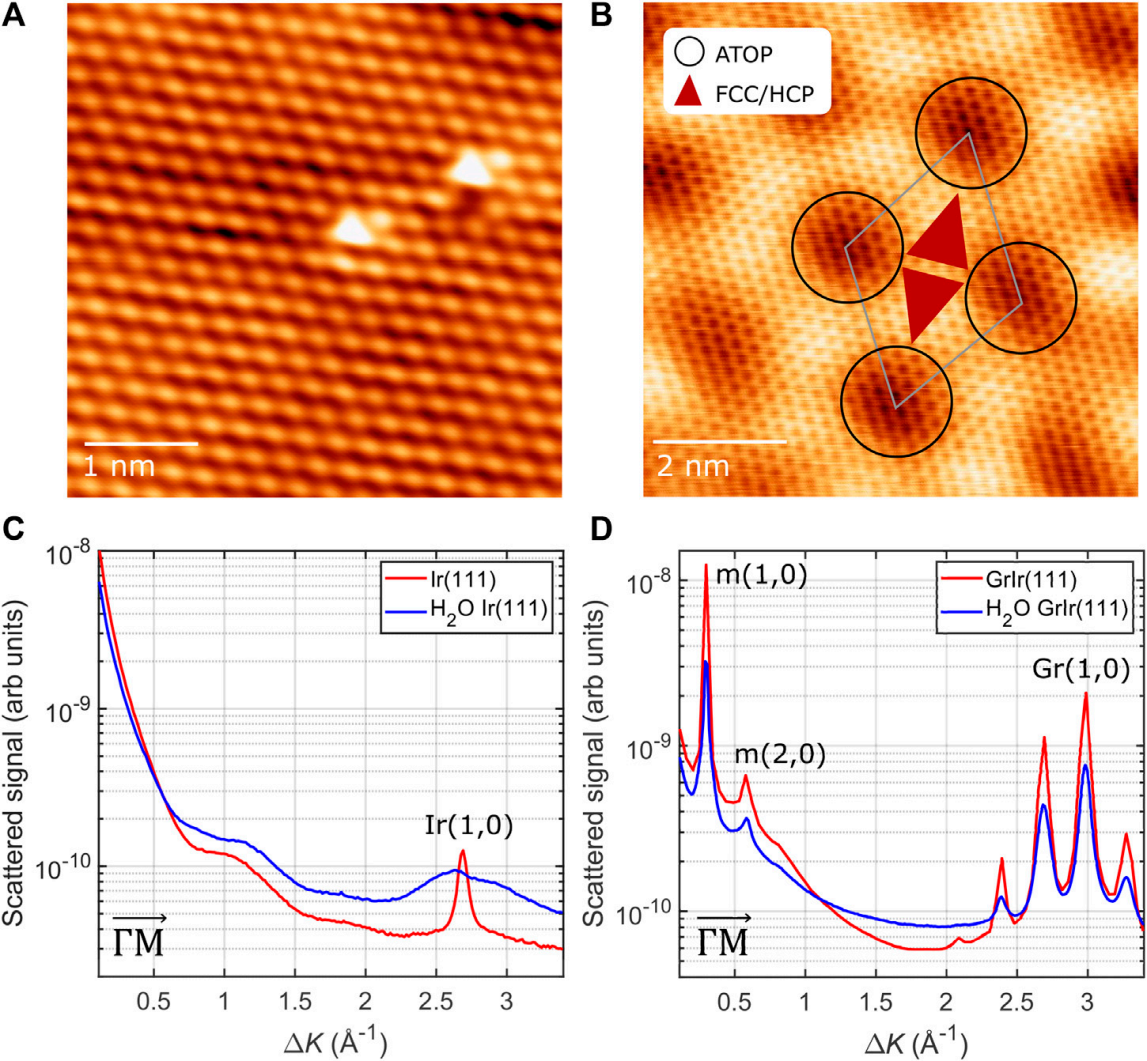
Top: STM images of **(A)** clean Ir(111) (Vt: 5.8 mV, It: 56.0 nA) and **(B)** graphene on Ir(111) (Vt: 7.6 mV, It: 0.94 nA); the schematic indicates the moiré unit cell with the repeating length scale of (25.02 ± 0.03) Å. The three high-symmetry regions of the moiré are as described in the main text. Bottom: diffraction scans from **(C)** a clean Ir(111) surface and **(D)** the GrIr(111) surface before (red) and after (blue) exposure to 0.1 L [Ir(111)] and 0.25 L [GrIr(111)] of water at 120 K. The diffraction peaks with the diffraction order are marked and labeled in the figure. The peaks arising from the moiré superstructure, labeled “m,” appear at low Δ*K* values and appear again around the first-order graphene peak, the latter labeled “Gr.”

Figures 1A, B show atomic-resolution STM images of Ir(111) and GrIr(111), respectively. The lattice constant of the Ir(111) surface was measured as (2.7 ± 0.1) Å, slightly larger than the lattice constant of graphene, which was measured as (2.5 ± 0.1) Å, in agreement with literature values (N’Diaye et al., 2008). This slight lattice mismatch gives rise to a moiré superstructure, visible as the large-scale repeating depressions in the STM image of GrIr(111) (Figure 1B). The moiré unit cell is schematically illustrated in Figure 1B. There are three high-symmetry regions: ATOP regions, where the center of a carbon hexagon sits directly on top of an Ir atom and are seen as dark depressions in the STM image (N’Diaye et al., 2008), and HCP and FCC regions, where every second carbon atom is positioned directly above an Ir atom with every other carbon atom in a bridge site. In the HCP regions, the carbon atoms in bridge sites lie above an Ir atom in the third surface layer, while in the FCC regions, the carbon atoms in bridge sites lie above an Ir atom located in the second layer. The bright protrusions in the STM image of the bare Ir(111) surface in Figure 1A indicate adsorbates, most likely oxygen atoms that remain chemisorbed following cleaning.

Diffraction patterns were recorded from both the Ir(111) and GrIr(111) substrates using HAS before and after exposure to the water dose that roughly corresponds to 0.07 ML of water at 120 K. The intensity of elastically scattered helium atoms versus the scattering momentum transfer, Δ*K*, along the $\bar{\Gamma M}$ direction is shown in Figures 1C, D. The Ir(111) surface gives rise to a single diffraction peak at Δ*K* = 2.69 Å^−1^ corresponding to a lattice spacing of (2.70 ± 0.03) Å equivalent to the Ir atom lattice. The diffraction pattern for the GrIr(111) sample shows peaks at Δ*K* = 0.30 Å^−1^ and Δ*K* = 3.00 Å^−1^, corresponding to lattice spacings of (25.02 ± 0.03) Å and (2.43 ± 0.03) Å, respectively. These values agree with the periodicities of the moiré lattice and graphene lattice measured with the STM (N’Diaye et al., 2008). All peak intensities are slightly reduced after water is adsorbed on the surface, indicating low water coverage. Water exposure on either surface did not result in any new peaks, indicating that no new lattice emerges following water adsorption.

The diffusion of H_2_O monomers adsorbed on Ir(111) and GrIr(111) was studied experimentally via the ^3^HeSE method by measuring the polarization of scattered ^3^He atoms after scattering from the substrate as a function of spin-echo time. The polarization gives the intermediate scattering function (ISF), *I*(Δ*K*, *t*), described by Eq. 1. Since ^3^He scattering is a surface-only effect, the ISF provides a measure of surface correlation on the length scale and direction given by the scattering momentum transfer, Δ*K*, after the spin-echo time *t* = *t*_SE_. Both parameters were varied in the experiment: *t*_SE_ was varied by adjusting the solenoid fields that spin-encode the ^3^He atoms; the Δ*K* direction was varied by adjusting the angle of the incident beam. For scattering from mobile species, the ISF can usually be written as

$$I\left(\Delta K,t\right)=I_{0}\left(\Delta K,0\right)e^{-\alpha \left(\Delta K\right)\cdot t}+C\left(\Delta K\right),$$ (1)

where *I*_0_ is the polarization measured at *t* = 0 and *C* is an offset reflecting persistent polarization caused by elastic scattering of ^3^He atoms from static defects on the substrate, i.e., vacancies or adsorbates. The change in the degree of correlation with spin-echo times is described by the dephasing rate, *α*(Δ*K*). The loss in correlation arises from ^3^He atoms that scatter quasi-inelastically from diffusing adsorbates and, when analyzed as a function of Δ*K*, provides information on the diffusing species in k-space.

**FIGURE 2:**
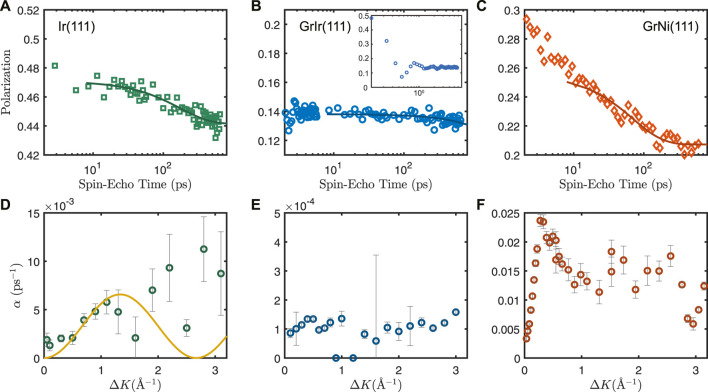
Top: Reduction in surface correlation as a function of spin-echo time following ^3^He scattering from approximately 0.07 ML water adsorbed at 125 K on **(A)** Ir(111), **(B)** GrIr(111), and **(C)** GrNi(111) at Δ*K* = 1 Å^−1^, measured in the $\bar{\Gamma M}$ direction. The data (unfilled symbols) were fitted with the ISF function (Eq. 1), resulting in the solid lines. Bottom: Dephasing rate, *α*, obtained from the ISF fit at each Δ*K* measurement in the $\bar{\Gamma M}$ direction is plotted for **(D)** Ir(111), **(E)** GrIr(111), and **(F)** GrNi(111). Uncertainty in *α* is given as the corresponding confidence bounds (1*σ*) of each exponential fit. Data shown in **(D)** can be fitted to the Chudley–Elliot model (Eq. 2), and the fit is shown with a solid line, allowing for jumps to the nearest neighbor only. Data presented in **(C,F)** for GrNi(111) were measured elsewhere and are adapted from the study by Tamtögl et al. (2021).

The results of typical H_2_O diffusion measurements on Ir(111) and GrIr(111) are presented in Figures 2A, B, respectively. Water was adsorbed to approximately 0.07 ML coverage at 125 K, and the polarization was measured along the $\bar{\Gamma M}$ direction as a function of spin-echo times from 0 to 642 ps at Δ*K* values from 0 to 3.1 Å^−1^. Figure 2C includes measurements for H_2_O dynamics on GrNi(111) collected by Tamtögl et al. (2021) using the same HeSE setup, and these data are included for comparison. In Figures 2A–C, the degree of polarization is plotted as a function of spin-echo times at Δ*K* = 1 Å^−1^. For GrIr(111), we ascribe the oscillating signal at spin-echo times below 8 ps to scattering from phonons. These data are shown as the inset in Figure 2B. The oscillating signals at short spin-echo times lack any characteristic dependence on Δ*K* and, while this initial decay could relate to vibrational or rotational motions within a unit cell, such effects would not alter the subsequent analysis or conclusions. The data for Ir(111) in Figure 2A extend to low spin-echo times, but as for data for GrIr(111), only the loss in correlation after 8 ps was fitted to Eq. 1 to obtain the dephasing rate, *α*. Dephasing rates were analyzed for Δ*K* in the range 0–3.1 Å^−1^, and the results are plotted in Figures 2D–F. Again, data for H_2_O dynamics on GrNi(111) were taken from the study by Tamtögl et al. (2021) and are included for comparison.

The data recorded from water adsorbed on Ir(111) show a tendency to decay at longer spin-echo times (Figure 2A), demonstrating quasi-elastic scattering from diffusing water molecules on the Ir(111) surface. The values for *α* obtained by fitting the ISF function in Eq. 1 at Δ*K* from 0 to 3.1 Å^−1^ are plotted in Figure 2D. In contrast, the data recorded from water adsorbed on GrIr(111) (Figure 2B) indicate no evidence of H_2_O translational diffusion on GrIr(111) across the spin-echo times accessible with this experiment. There was little loss of correlation for H_2_O on GrIr(111), on a timescale of hundreds of ps, at any Δ*K* value between 0 and 3.1 Å^−1^ across the temperatures investigated, from 120 to 160 K. Thus, we conclude that water should diffuse at a lower rate than can be assessed via our ^3^HeSE experiment. To set an upper limit for a loss of correlation, we assume that there is decay to some arbitrary point beyond the timescale accessible with the ^3^HeSE measurement. By setting this offset to half of the value of the last data point, we obtain *α* values on the order of 10^−4^ ps^−1^, plotted in Figure 2E. Changing the offset to 25% or 75% of the last data point does not change the order of magnitude of the *α* values. The values of *α* at ΔK = 1 Å^−1^ for each substrate are summarized in Table 1. The dephasing rates, *α*, for GrNi(111), 10^−2^ ps^−1^, are an order of magnitude larger than values for water diffusing on Ir(111), 10^−3^ ps^−1^, which, in turn, are an order of magnitude larger than the upper limit values for water diffusing on GrIr(111), 10^−4^ ps^−1^. This trend is already evident from visual inspection of the loss in correlation as a function of spin-echo times in Figures 2A–C.

**TABLE 1:** Experimentally determined diffusion parameters for water monomers on Ir(111), GrIr(111), and GrNi(111).

Diffusion parameters					
	*α* at Δ*K* = 1 Å^−1^ (ps^−1^)	*τ* (ps)	*D* (m^2^/s)	*E*_*a*_ (meV)	*α*_0_ (ps^−1^)
Ir(111)	$∼10^{-3}$	1,200 ± 300	(1.5 ± 0.4) × 10^−11^	90 ± 40	-
GrIr(111)	$∼10^{-4}$	-	$<10^{-12}$	-	-
GrNi(111)	$∼10^{-2}$	65 ± 3	(4.1 ± 0.2) × 10^−10^	60 ± 4	5 ± 1

The columns provide the following: the dephasing rate *α*, resident time *τ*, diffusion coefficient *D*, activation energy *E*_*a*_, and the Arrhenius prefactor *α*_0_. Values for GrIr(111) are provided as approximate upper limits. Standard deviation is found through error propagation. Values for GrNi(111) are taken from the study by Tamtögl et al. (2021).

The dephasing data obtained from H_2_O adsorbed on Ir(111) can be compared to the analytical Chudley–Elliot (CE) model, which is the simplest approach to describe molecular single-jump diffusion (Chudley and Elliott, 1961; Barth, 2000; Jardine et al., 2009). This model assumes that an adsorbate rests at time *τ* between jumps from one adsorption site to another. The model describes *α* as

$$\alpha \left(\Delta K\right)=\frac{2}{\tau }\underset{n}{\sum }p_{n}\sin^{2}\left(\frac{\Delta K\cdot j_{n}}{2}\right),$$ (2)

where each *n* represents a unique jump, represented by **j**_*n*_, the jump vector for that particular jump, and *p*_*n*_, the probability that an adsorbate will make that particular jump.

The CE model, described by Eq. 2, was applied to the *α* values plotted in Figure 2D for *n* = 1, i.e., for a jump to the nearest neighbor with *p*_*1*_ = 1. Increasing *n* > 1 did not improve the goodness of fit. The resulting fit, weighted towards lower Δ*K* values by the uncertainties of the data points, is shown as the solid line in Figure 2D. We estimate a residence time, *τ* = (1200 ± 300 ps), with a jump length ⟨*l*⟩ = (2.72 ± 0.03) Å, where the uncertainty was measured from the diffraction scan in Figure 1C. We assume that the water molecule sits on top of a surface Ir(111) atom as water molecules sit in atop positions on other close-packed transition metal surfaces (Carrasco et al., 2013). Water dissociation has been reported to be thermally activated on Ir(111) (Pan et al., 2011), and this may explain the absence of any decay in polarization in our experiments when the sample temperature was increased above 135 K. It may be that at these elevated surface temperatures, water molecules fragment to form smaller radical species, which may chemisorb to the Ir(111) surface.

Using the values of *τ* and ⟨*l*⟩ from Eq. 2, we can then calculate a value for the diffusion coefficient, *D*, using

$$D=\frac{1}{4\tau }\left\langle l^{2}\right\rangle ,$$ (3)

giving a diffusion coefficient of (1.5 ± 0.4) × 10^−11^ m^2^/s for water on Ir(111) at 125 K in the $\bar{\Gamma M}$ direction. It should be noted that in arriving at this value for the diffusion coefficient, we have used the simplest possible hopping model in the CE model, and we assumed that water molecules are non-interacting, which may not be the case at a coverage we can, at best, estimate to be roughly 0.07 ML. We can also approximate an upper limit for the diffusion coefficient for water on GrIr(111). For GrIr(111), the upper limits of the dephasing rates are one order of magnitude lower than what we report for water on Ir(111) and two orders of magnitude lower than the values reported for water on GrNi(111). We assume that this translates to a water residence time of at least one order of magnitude longer than for water on Ir(111), i.e., *τ* > 12 ns. With a residence time of this order and assuming that the jumping length stays in the Å length scale (similar to that for water hopping on GrNi(111)), we can approximate that the diffusion coefficient for water on GrIr(111) is $<10^{-12}$ m^2^/s.

If we assume that diffusion is an activated process, with an activation energy barrier *E*_*a*_, then the relationship between *α* values at the same Δ*K* measured as a function of temperature is modeled by the Arrhenius relation as follows:

$$\alpha =\alpha_{0}\exp\left(\frac{-E_{a}}{k_{B}T_{s}}\right),$$ (4)

where *α*_0_ is the pre-exponential factor describing the jump frequency, *k*_*B*_ is the Boltzmann constant, and *T*_*s*_ is the temperature of the surface.

**FIGURE 3:**
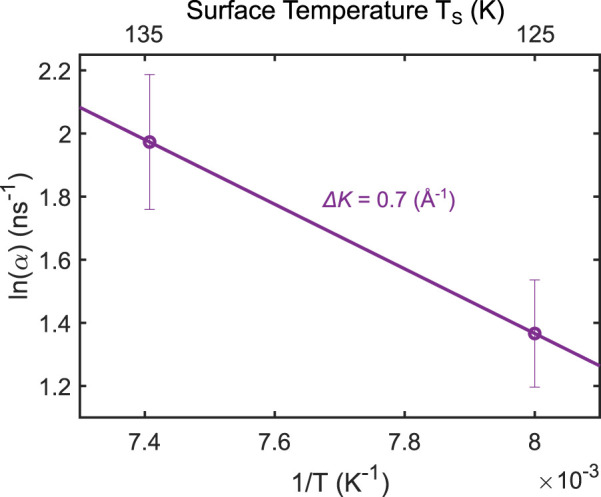
Arrhenius plot showing the temperature dependence of *α* at Δ*K* = 0.7 Å^−1^ for water on Ir(111). The error bars arise following error propagation.

Figure 3 shows an Arrhenius plot for *α* measured at 125 K and 135 K at ΔK = 0.7 Å^−1^. The data at 125 K were measured immediately after water deposition at this temperature, and data at 135 K were measured by annealing this sample to 135 K. Only data at these two temperatures were available in our experiments. The ΔK value of 0.7 Å^−1^ was chosen for the Arrhenius analysis because it provided the best signal-to-noise ratio in the experiment, with a low value of ΔK representing the jump to the nearest neighbor on the Ir(111) surface. The activation energy *E*_*a*_ can be extracted from the slope between these data points and is estimated to be (90 ± 40) meV. The large uncertainty of the measurement does not allow for an estimation of the exponential prefactor *α*_0_. Our value of *E*_*a*_ is similar to the barrier of (80 ± 8) meV, which is measured for water monomer diffusion on Cu(100) at temperatures below 30 K (Bertram et al., 2019). This is despite our ^3^HeSE measurements arising from water adsorbed at considerably higher temperatures, 125–135 K, demonstrating the veracity of the conclusion drawn by Bertram et al. that their value should be accurate across a large temperature range. An alternative approach to calculate the activation energy is to arbitrarily assume that the value of *α*_0_ is identical for water on Ir(111) and water on GrNi(111), i.e., 5 ps^−1^ (Tamtögl et al., 2021). This gives an activation energy of (77 ± 3) meV for Δ*K* = 0.7 Å^−1^ at 125 K, agreeing with the value found in Figure 3.

## 4 Discussion

**FIGURE 4:**
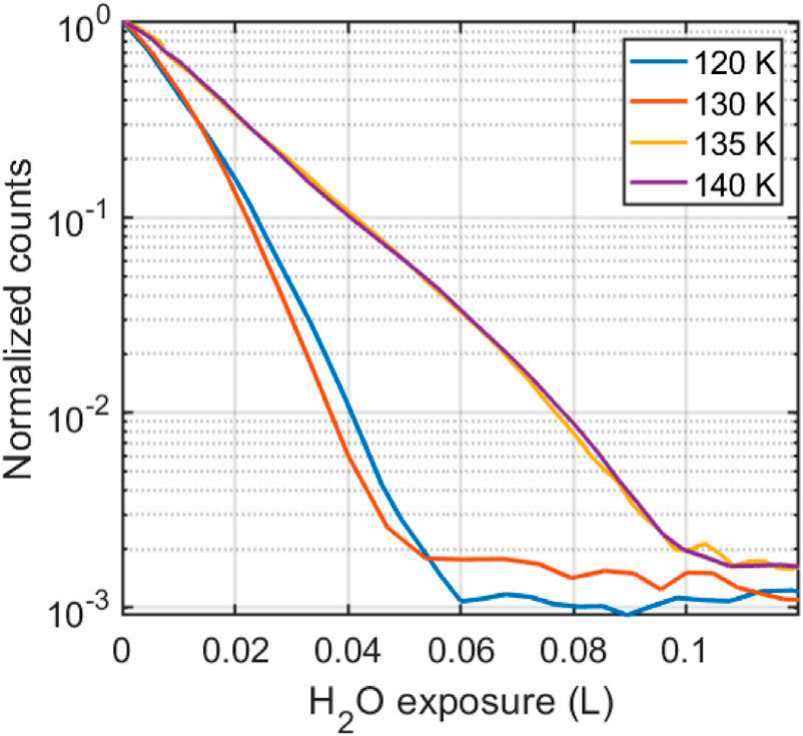
Helium scattering intensity at the specular angle, as a function of water fluence on GrIr(111), for sample temperatures in the range 120–140 K. The helium reflectivity signal decreases with an increase in water exposure, with a change in the rate of decrease between 130 and 135 K.

Our results demonstrate that if water is diffusing on GrIr(111), it must happen with a rate that is an order of magnitude lower than that of water diffusing on Ir(111) and two orders of magnitude lower than that of water diffusing on GrNi(111) (Tamtögl et al., 2021). Before discussing what might hinder water diffusion on GrIr(111), we first ask if we expect diffusion to occur in the temperature window studied, i.e., between 120 and 160 K. To this end, water was adsorbed onto the GrIr(111) substrate, and the helium reflectivity at the specular angle was recorded as a function of water exposure, at temperatures of adsorption between 120 and 160 K. The results, plotted in Figure 4, show a decrease in the intensity of elastically scattered helium atoms as a function of water exposure. The decrease results from the diffuse scattering of He atoms from water adsorbates on the GrIr(111) substrate as water molecules adsorb on the substrate. In all cases, the decrease is exponential, suggesting that adsorbates are isolated as they stick on the substrate (Farias and Rieder, 1998). There is minimal difference between the rate of decrease in the reflectivity between 120 and 130 K, indicating similar adsorption kinetics at these temperatures. The rate of decrease with an increase in exposure corresponds to the overlap of scattering cross-sections of the adsorbates on the surface. If the adsorbates “sit and stick”, the cross-sections will overlap, giving a simple relationship for the rate of reduction in the reflectivity signal. However, if the adsorbates repel or attract each other, that will increase or decrease the rate of loss in reflectivity, respectively (Farias and Rieder, 1998). There is a clear change in the rate at which reflectivity decreases when the substrate temperature increases from 130 to 135 K, with the reflectivity decreasing much slower at the higher adsorption temperatures. This low rate indicates an abrupt change in the adsorption kinetics at 135 K, which we attribute to either a sudden change in the sticking coefficient at 135 K or to the growth of a more ordered ice layer. Infrared reflection absorption spectroscopy experiments indicate that at low coverage, water adsorbates tend to aggregate into clusters at HCP regions on the GrIr(111) moiré, providing a confined environment for these water molecules and a distinctive IR spectrum (Gleißner et al., 2019). This interpretation concurs with results from low-temperature STM experiments (Standop et al., 2015). Infrared spectroscopy shows an annealing-induced structural change for water confined to HCP regions on GrIr(111) when the temperature is increased above 140 K (Gleißner et al., 2019). Increasing the surface temperature to 150 K led to water desorption (Supplementary Figure S1 in Supplementary Material), which agrees well with TPD measurements (Standop et al., 2015). The data in Figure 4 indicate that water gains some mobility across the temperature range in which ^3^HeSE measurements were recorded, specifically between 130 and 135 K, and one might expect the change in sticking coefficient and/or the structural rearrangement to coincide with a higher rate of water diffusion. Such diffusion was not observed in the ^3^HeSE data, indicating that if isolated water molecules do diffuse, they do so at a low rate, a rate much lower than the rate at which water diffuses on GrNi(111) (Tamtögl et al., 2021).

We note that the He reflectivity signal remained low at all temperatures below 150 K, even after extended waiting times, following water adsorption on the GrIr(111) substrate. This is in contrast to He scattering from water adsorbed on GrNi(111), where it was reported that the helium reflectivity signal recovered after water adsorption at 110 K and a diffraction pattern emerged (Tamtögl et al., 2021). Those authors attributed this increase in reflectivity to mobile water that migrated to form large ice clusters at 110 K. The moiré superstructure of GrIr(111) has been reported to lead to patterned adsorption of hydrogen atoms (Jørgensen et al., 2016), oxygen atoms (Cassidy et al., 2018; Kyrkjebø et al., 2021), metal clusters (N’Diaye et al., 2009; Feibelman, 2008), and water ice (Standop et al., 2015), with a slight preference for absorbates to be confined to the HCP regions of the moiré. Carbon atoms in the graphene basal plane have a registry with every second carbon atom above an Ir atom and lie closest to the Ir(111) substrate in the HCP regions, making the carbon atoms in those areas most readily available to form covalent bonds with the underlying Ir surface (Jørgensen et al., 2016). Standop et al. (2015) showed, with the LT-STM, that solid amorphous water became trapped in the HCP regions when adsorbed at temperatures below 80 K. Due to the patterned adsorption of water on GrIr(111), the surface was described as a pattern of hydrophilic regions in a hydrophobic matrix. In our experiments, we observed no measurable diffusion of water species, even at temperatures close to the water desorption temperature. We speculate, then, that water species on the GrIr surface are trapped in clusters at the HCP regions of the GrIr moiré. The high binding energies for adsorbates in these clusters at the HCP regions of the moiré might increase the activation energy for water monomer/dimer/trimer diffusion to a value comparable to that of desorption so that desorption competes with the diffusion of isolated water species.

Recently, it was shown, by measuring the freezing onset temperature of a water droplet on a cooled surface, that pristine and functionalized graphene grown on Ir(111) and Ru(0001) exhibit anti-icing properties (Akhtar et al., 2019; Kyrkjebø et al., 2021). Interestingly, graphene prepared on both of these surfaces gives rise to moiré superstructures, which are preserved after the adsorption of functional groups (Cassidy et al., 2018; Novotny et al., 2018). The freezing onset of a water droplet on GrIr(111) was reported as (−15 ± 3)°C and reduced to (−21 ± 1)°C after the introduction of chemisorbed oxygen on the GrIr(111) surface (Kyrkjebø et al., 2021). Kyrkjebø et al. (2021) proposed that a lower rate of ice nucleation occurred on the O-Gr/Ir(111) systems because interfacial water became more viscous in the presence of the chemisorbed oxygen (Zokaie and Foroutan, 2015). This increased viscosity provides a barrier to ice nucleation (Li et al., 2014). According to the crystal nucleation theory, ice growth becomes exothermic when a nucleus reaches a critical size, meaning that the initial nucleation step is rate-determining for ice growth. We speculate that the macroscopic anti-icing properties of the modulated graphene-based surfaces can be explained by the slow molecular diffusion of water reported here for water adsorbed on the GrIr(111) surface.

Our results demonstrate that the microscopic mechanism for water molecule diffusion on graphene is strongly substrate dependent, with the rate of water diffusion on GrIr(111) being at least two orders of magnitude lower than water diffusion on GrNi(111). Since water diffusion is faster on the bare Ir(111) surface than on the Gr/Ir(111) surface, we conclude that the graphene-Ir interaction determines the microscopic diffusion properties of single water molecules. Hence, tuning of the graphene-substrate interaction may provide a pathway to improve the de-icing properties of graphene films on metallic substrates.

## 5 Summary

Isolated water molecules on GrIr(111) are reported to diffuse at a rate with an approximate upper limit of 10^−12^ m^2^/s. This rate is at least one order of magnitude lower than that of isolated water molecule diffusion on Ir(111) and two orders of magnitude lower than that of water diffusion on GrNi(111). We propose that it is the graphene–metal interaction that determines the microscopic diffusion properties of single water molecules on the water–Gr/Ir(111) system. Specifically, the corrugated moiré superstructure of the loosely coupled GrIr(111) system can be viewed as a landscape of different binding energies, with water molecules binding more strongly at the so-called HCP regions, which may then hinder the diffusion of isolated species. Future research will aim to understand the nature of the interaction between water molecules and the graphene basal plane in these confined spaces at the HCP regions. Understanding the nature of this bond, i.e., chemisorption versus physisorption, may be important for hindering water diffusion on other potential graphene-based, anti-icing coatings.

## Data Availability

The datasets presented in this study can be found in online repositories. The data are archived on Zenodo with DOI: https://doi.org/10.5281/zenodo.8124927.
