# Supplementary material for: 3He spin-echo scattering indicates hindered diffusion of isolated water molecules on graphene-covered Ir(111)
Source: Front Chem. 2023 Oct 6;11:1229546. doi: 10.3389/fchem.2023.1229546 (PMC10587411; doi:10.3389/fchem.2023.1229546)
Supplement: Supplementary file 1 [file DataSheet1.PDF]

^{3}He spin-echo scattering indicates hindered diffusion of isolated water molecules on graphene-covered Ir(111)

Signe Kyrkjebø [1,2], Andrew Cassidy [1*], Sam Lambrick [3], Andrew Jardine [3], Bodil Holst[4], Liv Hornekær [1,2]
[1]Center for Interstellar Catalysis, Department of Physics and Astronomy, Aarhus University, 8000 Aarhus C, Denmark
[2]Interdisciplinary Nanoscience Center, Aarhus University, 8000 Aarhus C, Denmark
[3]Cavendish Laboratory, University of Cambridge, Cambridge, UK, CB3 0HE.
[4]Institute of Physics and Technology, University of Bergen, 5009 Bergen, Norway *Corresponding author Andrew Cassidy (amc@phys.au.dk)

SUPPLEMENTARY MATERIAL

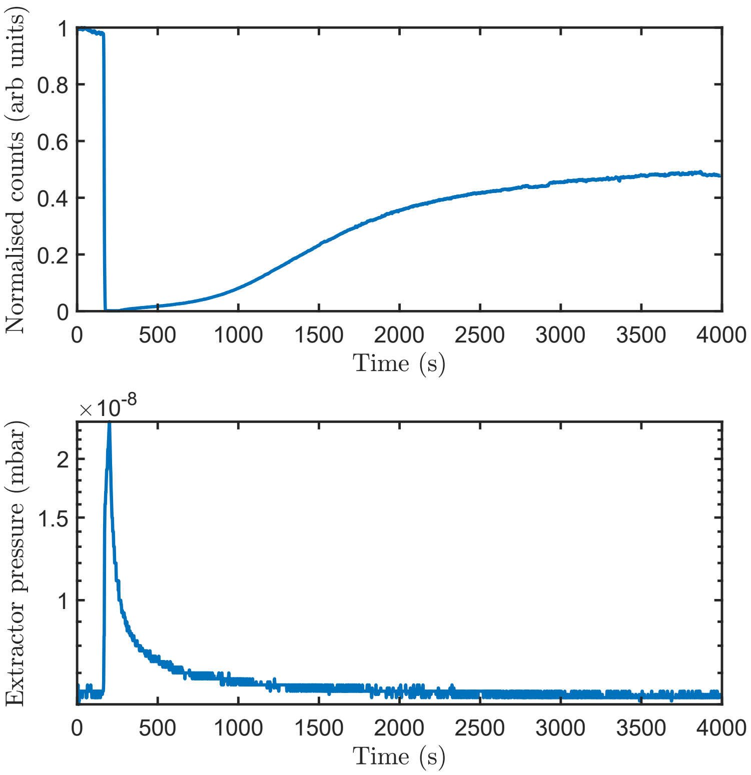

Figure S1. Water deposition measurements on GrIr(111) at 150 K. A graphene layer on the Ir(111) substrate was exposed to water at 150 K while the scatted He atom signal was recorded at the specular angle. The upper panel shows the helium scattering intensity at the specular angle as a function of time. The lower panel shows the partial pressure of water that the sample was exposed to, on the same time scale. The water dose starts at approx. 100 s and this gives rise to a sharp reduction in the intensity of scattered He atoms. The water partial pressure was reduced to nominal zero after approx. 1000 s and subsequently the He scattering signal recovered. This indicates that water desorbs from the surface at 150 K when the overpressure is reduced.
